# Evaluation of the Biocompatibility of CS-Graphene Oxide Compounds *In Vivo*

**DOI:** 10.3390/ijms20071572

**Published:** 2019-03-29

**Authors:** Diego López Tenorio, Carlos H. Valencia, Cesar Valencia, Fabio Zuluaga, Mayra E. Valencia, José H. Mina, Carlos David Grande Tovar

**Affiliations:** 1 Escuela de Odontología, Grupo biomateriales dentales, Universidad del Valle, Calle 13 No. 100-00, 76001 Cali, Colombia; dilote@hotmail.com (D.L.T.); carlos.humberto.valencia@correounivalle.edu.co (C.H.V.); 2 Laboratorio SIMERQO polímeros, Departamento de Química, Universidad del Valle, Calle 13 No. 100-00, 76001 Cali, Colombia; cesar.valencia@correounivalle.edu.co (C.V.); hector.zuluaga@correounivalle.edu.co (F.Z.); 3 Grupo de Materiales Compuestos, Escuela de Ingeniería de Materiales, Universidad del Valle, Calle 13 No. 100-00, 76001 Cali, Colombia; maelvaza@hotmail.com (M.E.V.); jose.mina@correounivalle.edu.co (J.H.M.); 4 Grupo de Investigación de Fotoquímica y Fotobiología, Universidad del Atlántico, Carrera 30 No. 8-49, 081008 Puerto Colombia, Colombia

**Keywords:** bone-regeneration, chitosan, graphene oxide, scaffolds

## Abstract

In the last few years, graphene oxide (GO) has gained considerable importance in scaffold preparation for tissue engineering due to the presence of functional groups that allow the interaction between the extracellular matrix and the components of the cellular membrane. The interaction between GO and chitosan (CS) can not only improve the biomechanical properties of the scaffold but also generate a synergistic effect, facilitating tissue recovery. *In vivo* studies on GO are scarce; therefore, biocompatibility tests on CS-GO scaffolds and bone regeneration experiments on critical size defects were carried out on *Wistar* rats. Scaffolds made of CS, CS-GO 0.5%, and CS-GO 1% were prepared and implanted on *Wistar* rats cranial bones for three months. Scaffold samples were analyzed through histochemistry and scanning electron microscopy. The analysis performed showed reabsorption of the material by phagocytic activity and new bone formation. The CS-GO 0.5% formulation gave the best performance in bone regeneration, with excellent biocompatibility. These results show the potential of this compound for tissue regeneration opening and medical applications.

## 1. Introduction

In recent years, the use of graphene oxide (GO) to reinforce scaffolds for tissue engineering has gained importance. Besides improving biomechanical properties, it can produce a synergistic effect that would improve biological properties when combined with other materials such as CS [1].

However, some doubts remain about the biocompatibility of GO in tissue engineering as there is not enough *in vivo* experimental evidence, and in *vitro* cytotoxicity results indicate that some side effects may occur.

It is clear that the systemic effects of a biomaterial on human beings’ health depend on some factors such as particle size, concentration, physicochemical properties, time of exposure, and route of entry into the organism. According to Peruzynska et al. [2], the biocompatibility of structures elaborated with graphene depend not only on dose and time but also on some chemical parameters such as layer number, shape, size, chemical functionalization, and surface characteristics.

In the case of GO, different investigations have been carried out to evaluate its effects on different types of cells and white organs, as well as to mitigate its cytotoxic effect.

In a review by Pinto et al. [3], it is reported that bacteria and mammalian cells decrease their viability when directly exposed to GO materials. *In vivo* studies showed high toxicity (approximately 50% of deaths), with accumulation in lung and liver and inflammation depending on dose and concentration. After a few hours of oral ingestion, there was an increase in these effects in the stomach and intestine but not inside other essential organs. After one day, some amounts were found in organs that disappear within a few days. Intratracheal introduction produced pulmonary inflammation and the subdermal route generating a slight inflammatory response. However, they also found that GO toxic effects decreased when it was incorporated into biomaterials [3,4].

Qu et al. [5] intravenously injected solutions of GO in buffer phosphate (PBS) in rats, confirming a toxic effect with accumulation mainly in the lungs. Dose-dependent cytotoxicity has been reported in human epithelial cells cultures [6].

In specialized literature, there is a consensus that GO cytotoxic effects occurred when there was direct contact with cells by physically damaging the lipid layer of the cell membrane [4] and increasing production of reactive oxygen species (ROS) [7], possibly with the initiation of apoptotic processes. However, some authors also found that this cytotoxic effect can be controlled by functionalization and binding to other compounds, which, in addition to improving biocompatibility, allow increased solubility and stability [4].

Awaja et al. [8] found low in *vitro* toxicity when coating polymers like polyether ether ketone (PEEK) and polyethylene terephthalate (PET) with GO. Wang et al. [9] demonstrated cytocompatibility and *in vivo* degradation of CS-GO scaffolds implanted in rat subdermal tissues. Xie et al. [10] found that incorporating GO into calcium phosphate (CaP) frameworks did not affect biocompatibility but instead improved surface characteristics that allowed a bone-inductive effect.

Our group previously reported the synthesis and characterization of CS-GO scaffolds by the freeze-drying method, having good results in the biocompatibility analysis in subdermal tissue regeneration after 30 days of implantation. It was clear that the material was biocompatible and that it did not generate an inflammatory response. However, more *in vivo* assays for critical size defects are necessary in order to obtain more information about the biocompatibility of the materials and their influence on the tissue regeneration processes, due to the fact that in this kind of defect, the tissue will not regenerate by itself, it will need some stimulation from the implanted materials.

For the above reasons, the objectives of this research were to evaluate the *in vivo* biocompatibility of Chitosan-graphene oxide (CS-GO) scaffolds and their potential in stimulating bone regeneration of critical size defects after three months of implantation in *Wistar’s* parietal bone.

## 2. Results

### 2.1. Characterization of CS, GO, and CS-GO Scaffolds

All this characterization was reported by our group elsewhere [11]. Briefly, chitosan was characterized using Fourier Transform Infrared Spectroscopy (FTIR) and Proton Nuclear Magnetic resonance spectroscopy (^1^H-NMR), in order to elucidate the structure of the molecule and its deacetylation degree (66.7%). Gel permeation chromatography was used to determine the average molecular weight of the polymer (6481.87 g/mol) [11].

Graphene oxide was characterized by FTIR, Raman, and X-ray diffraction spectroscopy. Finally, to study GO tridimensional and nanometric structure, Atomic Force Microscopy (AFM) and Dynamic Light Scattering (DLS) were used. CS-GO scaffolds were prepared and characterized by FTIR, TGA, and scanning electron microscopy (SEM).

### 2.2. Histological Observations on Control Samples

In the experimental designs with negative defect control, the objective of the empty defect or control defect is to validate the investigation. In this work, three samples used as control showed non-binding in the defect, i.e., the surgical preparation performed behaved as critical size, and the organism was not able to complete the healing by bone regeneration presenting a fibrous type of scarring.

**Figure 1:**
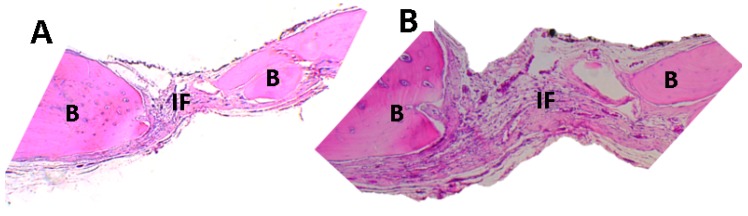
Control site (empty defect). Image (**A**) at 5× image (**B**) at 10× preparation zone. (**B**): peripheral bone, FI: a fibrous interface. Hematoxylin-eosin (HE) stain was used for the analysis of the preparation area.

**Figure 2:**
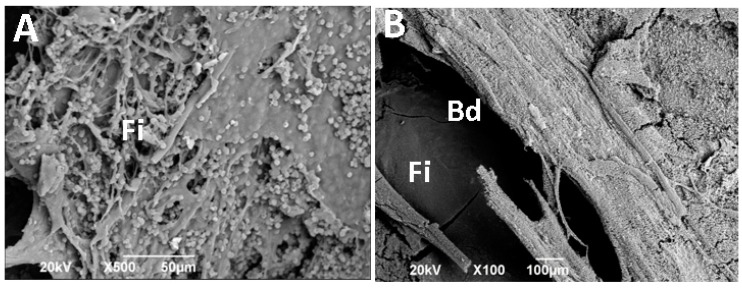
Scanning electron microscopy (SEM) image of the control site. Image (**A**) corresponds to the periosteal surface, and image (**B**) corresponds to the view from the meningeal surface. FI: Fibrous interface, BD: Bone defect.

Figure 1 and Figure 2 correspond to a sample of this group in preparations with hematoxylin-eosin stain (HE). It is apparent how the fibrous material (fibrous interface (If)) occupies the preparation area, and old bone areas (peripheral bone (B)) are in the process of remodeling.

Figure 2 corresponds to an image obtained by scanning electron microscopy in the preparation area. Here, it is notorious how the defect is covered by a material of fibrous appearance (If). Image A corresponds to the surface in contact with the periosteum. At the left end of the image, there is a preparation area corresponding to the defect. Image B corresponds to the same surface seen from the meningeal side, where it is clear that the deficiency remains without regeneration.

The previous observations are expected for this type of design, similar to reports in the literature. Some reports mention scarring of fibrous nature with parallel collagen fibers that seem to continue with the periosteum, with a thickness lower than the original bone and with evidence of regeneration in the edges of the preparation area [12,13,14,15].

### 2.3. Histological Observations in Experimental Samples

In general, all samples showed a similar behavior with partial degradation/resorption of the materials and replacement by newly formed bone tissue. The staining techniques used corresponded to hematoxylin-eosin (HE), Masson trichromacy (TM), and Gomori trichromacy (TG).

**Figure 3:**
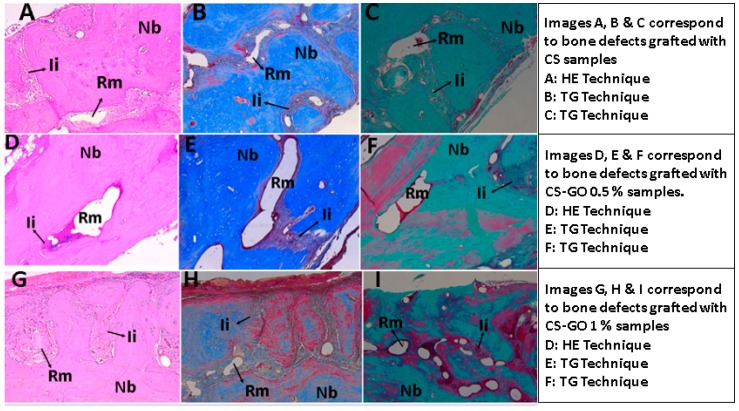
Experimental samples with images at 10×. Nb: Newly formed bone, Rm: Remnant material, Ii: Inflammatory infiltrate.

Figure 3 corresponds to 10× images of samples implanted with the three types of material, newly formed areas plus areas with remaining material, and regions in degradation/reabsorption processes are observed.

**Figure 4:**
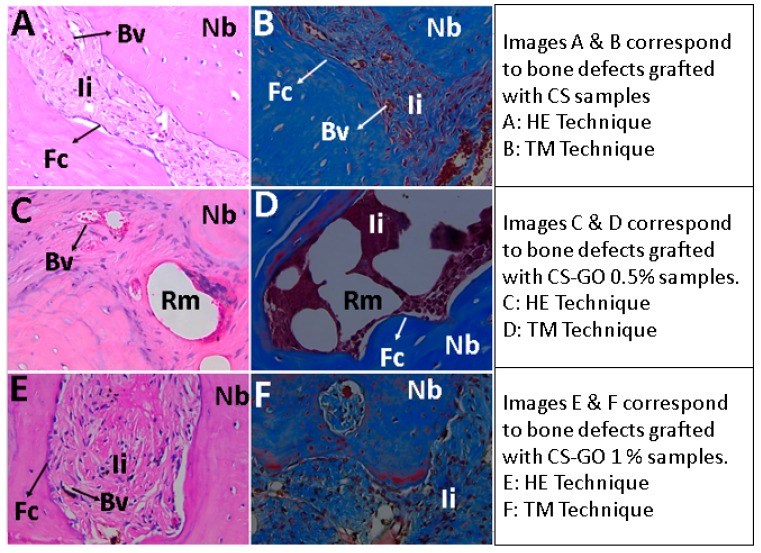
Experimental samples with images at 40×. Nb: Newly born bone, Ii: Inflammatory infiltrate, Rm: Remnant material, Fc: Fibrous capsule, Bv: Blood vessel.

At higher magnification, it is possible to observe particles’ remaining material surrounded by a fibrous capsule which is phagocytized by cells of a mixed inflammatory infiltrate, with the presence of abundant blood vessels (Figure 4).

From histological images taken at 4×, a histomorphometrically analysis was performed to determine the area of newly formed bone and the areas occupied with collagen I and collagen type III. The three groups were made up of three specimens each. The area of bone formation was greater in the group of specimens treated with CS-GO 0.5% with an average area formed of 95.287 ± 6.126%, as compared to 52.79% for those implanted with CS-GO at 1%. This previous result is statistically significant at the significance level of 0.1 (*p* = 0.0509). The post-hoc test showed differences between CS vs. CS-GO 0.5% (*p* = 0.005) and between CS-GO 0.5% vs. CS-GO 0.1% (*p* = 0.028).

SEM and dispersive energy spectroscopy (EDS) analyses showed differences in the surface appearance of the implanted area and the contents of calcium and phosphorus.

**Figure 5:**
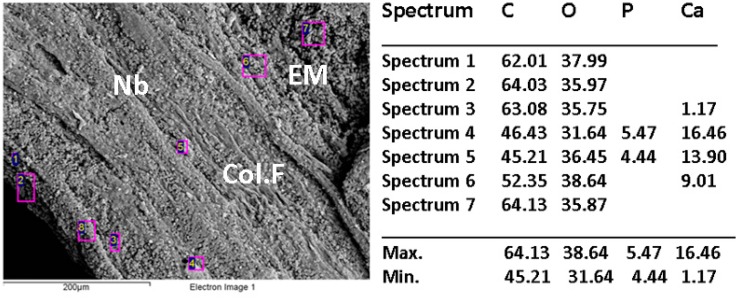
Scanning electron microscopy (SEM)/dispersive energy spectroscopy (EDS) analysis of control sample implanted in critical size defects in rat parietal bone *Wistar*. Nb: Newly born bone. EM: Extracellular matrix. Col-F: collagen fiber.

**Figure 6:**
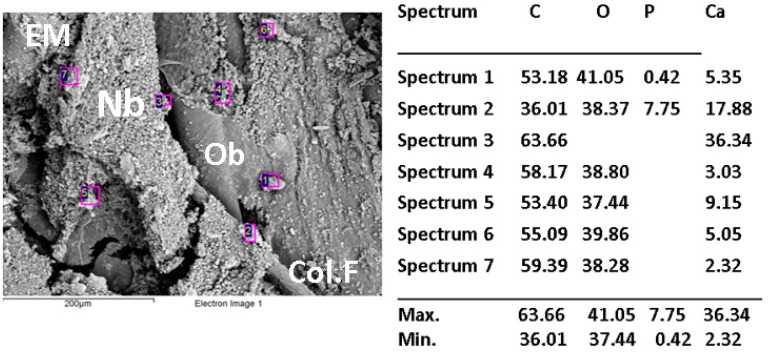
Scanning electron microscopy (SEM)/dispersive energy spectroscopy (EDS) analysis of CS-GO 0.5% sample implanted in critical size defects in rat parietal bone Wistar. Nb: Newly born bone. EM: Extracellular matrix. Col-F: collagen fiber. Ob: Osteoblast.

**Figure 7:**
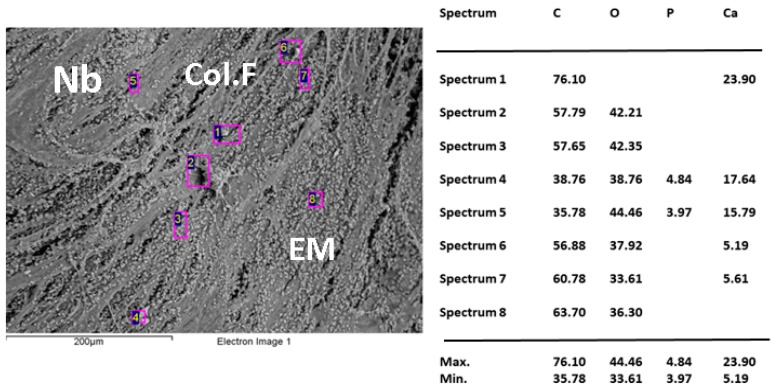
Scanning electron microscopy (SEM)/dispersive energy spectroscopy (EDS) analysis of CS-GO 1% sample implanted in critical size defects in rat parietal bone *Wistar*. Nb: Newly born bone. EM: Extracellular matrix. Col-F: collagen fiber.

Figure 5, Figure 6 and Figure 7 show the images of the three samples implanted with the different materials, the presence of structures compatible with collagen fibers, cells, and extracellular matrix in different proportions are observed.

The elemental analysis by EDS indicates that the sample CS-GO 0.5% presented the highest percentages of calcium and phosphorus.

## 3. Discussion

In this investigation, the differences in the results obtained are a consequence of the design of the experiment itself (critical defect with negative defect control), i.e., differences in defects (regeneration of the preparation) are due to the influence that the three formulations had on the repairing cells.

If we review the overall percentages of bone-formation in the three defects, the next observations arise: 34.19% bone formation for those implanted with CS, 95.28% for those embedded with CS-GO 0.5%, and 52.79% for those implanted with CS-GO 1%. A considerable difference arises between the scaffolds with GO and the ones that were made of CS alone. It is also notorious that CS with 0.5% GO was more effective in stimulating bone regeneration than the other two.

When comparing the results of the three groups in relation to the area of newly formed bone, statistically significant differences were found at 0.1, with a value very close to 0.05 (*p* = 0.05090) and with statistically significant differences in the post-analysis with comparison between CS vs. CS-GO 0.5% (*p* = 0.005) and between CS-GO 0.5% vs. CS-GO 0.1% (*p* = 0.028).

We explain these results between defects grafted with CS versus those grafted with GO through a chitosan-GO synergistic effect. Regarding the difference between GO 0.5 and GO 1%, the higher concentration of the GO seems to encourage a greater inflammatory response, which delays the healing process

The statistical results that show the 0.5% CS-GO formulation as the one with the highest new bone-formation area agree with the SEM images that present the samples of this formulation with a better level of development of the extracellular matrix, and with the elemental analyses (EDS) that show higher percentages of calcium and phosphorus in the sample (Figure 5, Figure 6 and Figure 7).

The lack of homogeneity in the analysis of the samples is due to bone mineralization, which is not uniform and varies in each specific site. Besides that, the fact that the values obtained by EDS correspond to particular locations means a high number of areas of analysis was selected across the same sample.

When reviewing the values, it is observed that the highest amounts of Ca and *p* were obtained for the sample of CS-GO 0.5%, indicating a more advanced mineralization process (bone formation).

Currently, the combination of CS and GO is considered one of the most promising nanocomposites in tissue engineering, due to the synergistic effect between CS (a material with recognized biocompatibility) and the excellent mechanical properties of GO [16], which would explain the superiority in the biocompatibility, mechanical, and thermal stability of the CS-GO nanocomposites compared to scaffolds of CS only. 

The hydrogen bonding interaction between the two compounds has allowed a better resistance material with different applications today, with the ability to positively modulate cell activity and cell regeneration [1].

It is proposed that GO beside promoting the adhesion and proliferation of osteoblasts, thanks to its high conductivity, can electrically stimulate osteoblasts during matrix formation [16,17]. For example, in a previous study conducted by Lee et al. (2011) [18], it was found that GO promoted stem cell differentiation. This observation was also pointed by Ming et al. in 2014 [19] and Dubey et al. (2015), highlighting graphene’s ability to stimulate osteoblastic differentiation [20].

Concerning the presence of collagen type I and III, there are no statistical differences between the three formulations, with the 0.5% CS-GO formulation having the highest percentages. Also, there are no differences in the portions of the two collagens. The presence of the two collagens in such similar rates indicates the presence of a young extracellular matrix, in a mineralization process.

In all cases of implantation, both CS and CS-GO are degraded by a mixed inflammatory infiltrate, surrounded by a fibrous capsule compatible with a chronic inflammatory response strong evidence of biocompatibility.

## 4. Experimental

### 4.1. Materials

*Aspergillus niger* mycelium was obtained from Sucroal (Cali, Colombia). All other reagents were obtained from Sigma-Aldrich (Palo Alto, CA, USA). CS was extracted and characterized previously (yield: 11%) [11]. GO was synthesized following Marcano. D. et al. methodology, obtaining 5 g of GO [21]. All the characterization was reported previously [11]. CS-GO scaffolds were prepared and characterized according to our previous report [11].

### 4.2. In Vivo Biodegradation

Scaffolds made of CS, CS with GO at 0.5% (CS-GO 0.5%), and CS with 1% GO (CS-GO 1%), were reduced to particles of irregular shape and size in a range of 200 to 600 µm and implanted in cranial defects of critical size in 16 *Wistar* adult male rats (5 mm diameter and 0.8 mm thickness). Biomodels were obtained from the Bioterio of the Universidad del Valle. As a control, four biomodels were left without implant (control by empty defect).

A group of twelve biomodels underwent histological analysis with hematoxylin-eosin (HE) and histochemistry (Masson trichromacy and Gomori trichromacy); the second group consisted of four biomodels which were analyzed by SEM and elemental analysis by dispersive energy spectroscopy (EDS).

After three months of implantation, euthanasia was performed with excess isoflurane®, and samples were recovered. The samples for immunohistochemistry technique were fixed in buffered formalin, dehydrated in alcohol solutions of ascending concentration (70%, 80%, 95%, and 100%), diaffinized with xylol and infiltrated with paraffin for later cutting at 5µm using a Thermo ScientificTM Histoplast Paraffin ™ and a Autotechnicon Tissue Processor ™ (Leica Microsystems, Mannheim, Germany). The samples were processed for histological analysis by hematoxylin and eosin and Masson trichromacy techniques. For the analysis of the images, a Leica DM 750 microscope with a Leica DFC 295 camera and Leica Application Suite version 4.12.0 (Leica Microsystems, Mannheim, Germany) imaging software was used 

The samples for SEM/EDS were dehydrated in ascending alcohols and analyzed through scanning electron microscope (JEOL JSM-6490LA, Musashino, Tokyo, Japan). Each sample was coated with a copper bath.

This research was reviewed, endorsed, and supervised by the Institutional Ethics Review Committee with experimental animals of the Universidad del Valle (CEAS 001-016).

### 4.3. Statistical Design

4× histological images were used to perform a histomorphometrical study to determine the formation of bone tissue in the defects created. The Image J program 1.3, NIH, was used to analyze the images.

The statistical analysis consisted of calculating measures of central tendency and dispersion for the variables of area of bone formation in percentage and presence of collagen type I and type III, for each of the intervention groups: CS (CS), CS plus 0.5% GO (CS-GO 0.5%), and CS plus 0.1% GO (CS-GO 0.1%).

Assumptions of normality were verified by the Shapiro–Wilk test and equality of variances with the Levene test. The contrast between groups to determine differences between distributions was performed using the nonparametric Kruskal Wallis test, and Bonferroni’s post-test was used in case of statistical significance. The level of confidence was set at 95% and of significance at 0.1 and 0.05. All estimates were conducted in STATA IC 15 (StataCorp LLC).

## 5. Conclusions

This study shows how materials based on chitosan and chitosan/graphene oxide are biocompatible, and its elimination in a living organism occurs by a foreign body reaction process where the content is fragmented and phagocytosed by the cells. Bone formation was simultaneous with the absorption of the scaffold material.

The CS-GO 0.5% formulation showed significant differences in terms of the ability to stimulate bone regeneration in critical size defects, maintaining adequate levels of biocompatibility, which allows to propose it as an election material in tissue engineering.

Implanted samples of Chitosan and graphene oxide 0.5% presented an advanced extracellular matrix development and with higher percentages of phosphorus and calcium, essential components of the extracellular bone matrix in the mineralization process.

The collagen distribution was similar for type I and II collagen, which indicated the presence of new bone tissue, (Woven bone). All these results show that the elaboration of scaffolds of chitosan reinforced with 0.5% graphene oxide is a good alternative in the regeneration of bone defects in critical size defects.
